# Assessing Pancreatic Enhancement Variability in ICU Patients with CT Hypoperfusion Complex: A Retrospective Single-Center Study

**DOI:** 10.3390/diagnostics16172808

**Published:** 2026-09-01

**Authors:** Ana-Maria Ungureanu, Bogdan-Florin Godje, Anamaria Alec, Daniel Malița, Sergiu-Ciprian Matei

**Affiliations:** 1 Department XV, University Clinic of Radiology and Medical Imaging, “Victor Babeș” University of Medicine and Pharmacy, Eftimie Murgu Square, No. 2, 300041 Timișoara, Romania; ungureanu.ana-maria@umft.ro (A.-M.U.); malita.daniel@umft.ro (D.M.); 2 Department of Radiology and Medical Imaging, “Pius Brînzeu” Emergency County Hospital, 300723 Timișoara, Romania; anamaria.alec@umft.ro; 3 Department of Doctoral Studies, “Victor Babeș” University of Medicine and Pharmacy, Eftimie Murgu Square, No. 2, 300041 Timișoara, Romania; 4 1’st Surgical Department, “Pius Brînzeu” Emergency County Hospital Timisoara, 300723 Timișoara, Romania; matei.sergiu@umft.ro; 5 Abdominal Surgery and Phlebology Research Center, “Victor Babeș” University of Medicine and Pharmacy, Eftimie Murgu Square, No. 2, 300041 Timișoara, Romania

**Keywords:** CT hypoperfusion complex, shock pancreas, pancreatic enhancement, contrast-enhanced computed tomography, intensive care unit, hypovolemic shock, septic shock

## Abstract

**Background**: The CT hypoperfusion complex describes a constellation of abdominal vascular, visceral, and parenchymal imaging signs occurring in severe systemic hypotension. Among these findings, pancreatic parenchymal enhancement remains controversial, with both hyperenhancement and hypoenhancement described in the literature. **Methods**: We performed a retrospective single-center analysis of contrast-enhanced abdominal CT examinations obtained between 1 October 2023 and 31 December 2024 in ICU patients with a clinically established diagnosis of shock. Of 266 patients identified using predefined shock-related ICD-10 codes, 94 underwent abdominal CT, and 44 unique patients fulfilled the imaging criteria for CT hypoperfusion complex, with one examination included per patient. Pancreatic attenuation was assessed across arterial, portal venous, and delayed phases and categorized relative to hepatic and splenic parenchyma as hyperenhancing, hypoenhancing, or isoenhancing. Enhancement categories were correlated with available clinical outcome and laboratory data. **Results**: The final cohort included 44 unique patients, each contributing one CT examination (mean age 59.5 ± 21.2 years; median 66 years; 28/44 women, 63.6%). Abnormal pancreatic enhancement was observed in 31/44 cases (70.5%): 20/44 (45.5%) showed hyperenhancement or mixed hyperenhancing patterns, 11/44 (25.0%) showed hypoenhancement without hyperenhancement, and 13/44 (29.5%) showed isoenhancement in all evaluable phases. In-hospital outcome data were available for 30/44 patients (68.2%). Among patients with known outcomes, mortality was 20/30 (66.7%). In an exploratory complete-case analysis, mortality was higher in patients with abnormal pancreatic enhancement than in those with isoenhancement (15/18, 83.3%, versus 5/12, 41.7%; Fisher’s exact *p* = 0.045; OR 7.0, 95% CI 1.29–37.91). The highest observed mortality proportion was found in the hyperenhancement/mixed group (9/10, 90.0%); however, the global comparison across the three pancreatic enhancement categories did not reach statistical significance (Fisher–Freeman–Halton exact *p* = 0.064). Therefore, this observed high proportion should be interpreted descriptively and does not establish that the hyperenhancement/mixed group had the worst prognosis. In addition, outcome availability differed according to pancreatic enhancement category, and selection bias cannot be excluded. **Conclusions**: Pancreatic enhancement variability was common among ICU patients with CT hypoperfusion complex. The observed association between abnormal pancreatic enhancement and all-cause in-hospital mortality was derived from an exploratory, unadjusted complete-case analysis and may reflect the severity of the underlying shock. Because outcome data were missing for 31.8% of patients and outcome availability differed according to pancreatic enhancement category, selection bias cannot be excluded. These findings do not establish a causal or independent prognostic role for pancreatic enhancement.

## 1. Introduction

In 1987, Taylor et al. introduced the term “hypoperfusion complex” to describe a characteristic group of abdominal CT findings observed in children with hypovolemic shock after trauma [1]. The concept has since expanded beyond pediatric trauma and is now used to characterize a recognizable CT pattern of severe systemic hypotension and impaired effective circulating volume in both traumatic and non-traumatic patients.

Shock is a life-threatening state of inadequate tissue oxygen delivery and utilization. Hypovolemic and hemorrhagic shock are typically driven by loss of intravascular volume and oxygen-carrying capacity, whereas septic shock combines circulatory, cellular and metabolic derangements related to dysregulated host response to infection [2,3]. In intensive care settings, these mechanisms may coexist, and imaging is frequently performed in clinically unstable patients to identify the underlying cause of deterioration and to detect secondary signs of profound hypoperfusion.

The CT hypoperfusion complex, also referred to in the older literature as the CT hypotension complex or hypovolemic shock complex, is not limited to traumatic hemorrhage. It has been described in patients with sepsis, cardiac arrest, cardiogenic shock, severe head or spinal injury, diabetic ketoacidosis and other causes of severe hypotension [4,5]. Therefore, radiologists evaluating abdominal CT examinations in ICU patients should actively search for this pattern, especially when the clinical indication is non-specific, or the hemodynamic severity is not fully conveyed on the request form.

Typical CT manifestations include reduced aortic and inferior vena cava caliber, an inferior vena cava “halo” sign, fluid-filled and thickened bowel loops with mucosal hyperenhancement, mesenteric hyperenhancement, adrenal hyperenhancement, abnormal renal enhancement, hypoenhancement of the liver or spleen, ascites and peripancreatic fluid [1,4,5,6]. In many descriptions, the diagnosis is supported by the coexistence of at least two vascular, visceral or parenchymal signs rather than by one isolated finding [5].

Among parenchymal findings, pancreatic appearance is particularly debated. The original pediatric trauma descriptions emphasized intense pancreatic enhancement as part of the hypoperfusion complex [1,7]. In adults, however, peripancreatic fluid and variable pancreatic enhancement have also been emphasized, and dynamic CT studies have suggested that early-phase pancreatic perfusion may decrease while delayed or prolonged enhancement may occur in later phases [8,9]. This variability creates uncertainty regarding how “shock pancreas” should be interpreted and whether it has prognostic value.

The aim of this study was to describe pancreatic enhancement patterns on multiphasic contrast-enhanced abdominal CT in ICU patients with CT hypoperfusion complex and to explore their unadjusted association with all-cause in-hospital mortality among patients with known outcomes. The study was not designed to assess causality or independent prognostic performance.

## 2. Materials and Methods

### 2.1. Study Design and Patient Selection

This retrospective single-center observational study reviewed contrast-enhanced abdominal CT examinations performed in ICU patients between 1 October 2023 and 31 December 2024. The institutional database was queried for all patients assigned ICD-10 diagnostic codes corresponding to cardiogenic shock (R57.0), hypovolemic shock (R57.1), septic shock (R57.2), other shock (R57.8), unspecified shock (R57.9), or traumatic shock (T79.4). All records matching these predefined codes during the study interval were screened, without sampling or selective enrolment. Shock had been diagnosed by the treating clinical team before CT based on the clinical presentation and routinely available laboratory and hemodynamic information. CT findings were not used to establish the clinical diagnosis of shock. Because of the retrospective design and the inclusion of different shock etiologies, a single study-specific numerical threshold for blood pressure, lactate, or vasopressor requirement was not imposed retrospectively. The manuscript was prepared following the general principles of STROBE reporting for observational studies.

**Figure 1:**
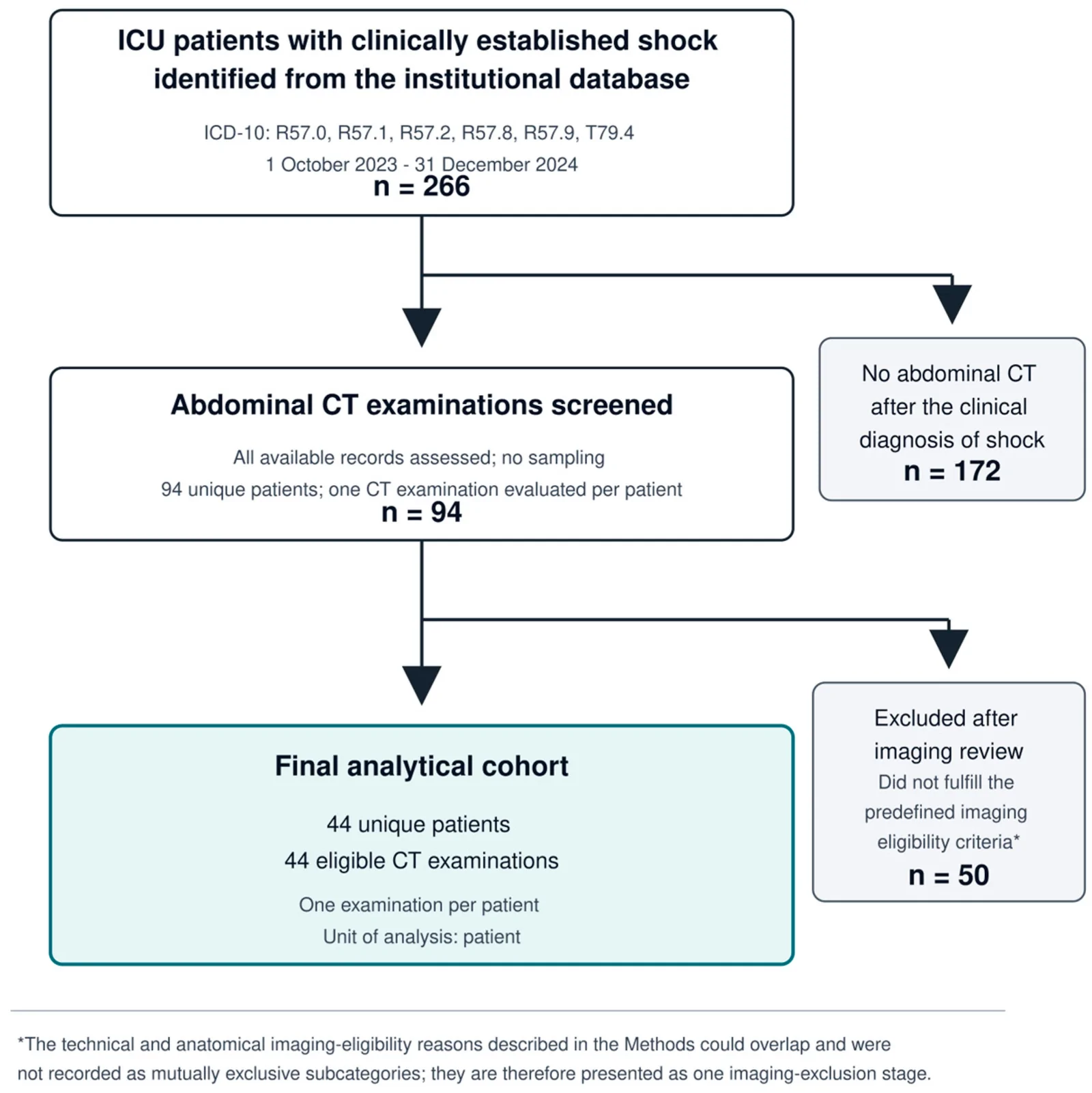
STROBE-style flow diagram of patient identification, CT examination screening, and construction of the final analytical cohort.

The database search identified 266 patients with a clinically established diagnosis of shock. Of these, 172 did not undergo abdominal CT and were therefore not eligible for imaging assessment. Abdominal CT examinations from the remaining 94 patients were screened according to predefined imaging and technical eligibility criteria. Fifty examinations did not fulfil the overall imaging definition of CT hypoperfusion complex, whereas 44 fulfilled the criteria and allowed pancreatic enhancement assessment. The technical and anatomical reasons for imaging non-eligibility could overlap and were therefore not treated as mutually exclusive sequential categories. The final cohort comprised 44 unique patients, each contributing one eligible CT examination. The patient was defined as the unit of analysis (Figure 1).

Patients were included if abdominal CT showed features compatible with the CT hypoperfusion complex and if at least arterial and portal venous phase images allowed assessment of pancreatic parenchymal enhancement. The working CT diagnosis required at least two compatible vascular, visceral or parenchymal findings, including reduced aortic or inferior vena cava caliber, inferior vena cava halo sign, abnormal bowel wall thickening or mucosal hyperenhancement, adrenal hyperenhancement, abnormal renal enhancement, hepatic or splenic hypoenhancement, peripancreatic fluid or ascites [4,5,10,11,12,13].

Exclusion criteria were non-contrast CT only, severe motion or beam-hardening artifacts preventing parenchymal assessment, major traumatic disruption of the pancreas, liver or spleen precluding representative ROI placement, pancreatectomy, focal pancreatic tumor replacing the measurement region and incomplete records preventing verification of the clinical diagnosis of shock or other essential eligibility variables. Patients with a confirmed clinical diagnosis of acute pancreatitis, characteristic imaging findings of acute pancreatitis, or pancreatic necrosis were excluded. The available clinical records were reviewed for a documented diagnosis of acute pancreatitis, compatible symptoms and elevated serum amylase or lipase levels when available. No patient included in the final analytical cohort had a confirmed diagnosis of acute pancreatitis. Cases with limited peripancreatic fluid were retained when representative pancreatic parenchymal ROI placement remained feasible and when no additional clinical, laboratory or radiologic findings supported acute pancreatitis, because peripancreatic fluid itself is a recognized manifestation of the CT hypoperfusion complex.

### 2.2. CT Protocol and Image Evaluation

Each patient underwent conventional spiral abdominal CT including unenhanced imaging followed by intravenous contrast-enhanced acquisitions. Examinations were performed using a CT scanner with 128 detector rows. A fixed volume of 125 mL of nonionic iodinated contrast material (350 mg I/mL) was administered intravenously at 2.5 mL/s, followed by a saline flush. Arterial-phase images were acquired at approximately 10 s after injection, portal venous-phase images at approximately 40 s, and delayed-phase images at approximately 180 s when clinically feasible. The acquisition parameters were 120 kVp, 600 mA, and contiguous 1.25 mm axial reconstructions. The delayed phase was not acquired or was not evaluable in four examinations.

The aortic diameter was measured anteroposteriorly above and below the renal arteries; a markedly reduced caliber (<12 mm) was considered supportive of a hypoperfusion complex in the appropriate clinical-imaging context. Inferior vena cava flattening was considered present when the anteroposterior diameter was <9 mm in consecutive segments or when low attenuation pericaval fluid produced an IVC halo sign. Bowel criteria included wall thickening greater than 3 mm, submucosal edema, fluid-filled loops and mucosal hyperenhancement relative to adjacent skeletal muscle. Parenchymal criteria included adrenal hyperenhancement, abnormal renal enhancement, hepatic or splenic hypoenhancement and peripancreatic fluid [4,5,10,11,12,13,14].

Images were reviewed on PACS/workstation using axial images, with multiplanar reconstructions available when required. In each acquisition phase, circular ROI(s), with an approximate area of 37 mm^2^, were placed within visually homogeneous pancreatic parenchyma at the level of the head/body/tail. ROI placement avoided the pancreatic duct, organ margins, major vessels, calcifications, focal lesions, visibly necrotic areas and areas affected by partial-volume averaging. ROIs were manually repositioned to sample the same anatomical region whenever technically possible. When multiple pancreatic ROIs were obtained, their mean was used for analysis.

Reference ROIs were placed in homogeneous areas of the right hepatic lobe and splenic parenchyma, avoiding vessels, focal lesions, artifacts and organ margins. Reference attenuation was defined as the mean of the hepatic and splenic measurements. Pancreatic and reference-organ attenuation values were obtained from the same acquisition phase.

Image assessment was performed by one radiologist with 20 years of experience in abdominal imaging. The reader was blinded to laboratory results and clinical outcome at the time of pancreatic attenuation assessment. Because measurements were performed once by a single radiologist, formal interobserver and intraobserver agreement was not evaluated.

For each phase, the attenuation difference was calculated as pancreatic attenuation minus reference-organ attenuation. The pancreas was classified as hyperenhancing when this difference was greater than +20 HU, hypoenhancing when it was lower than −20 HU, and isoenhancing when it ranged from −20 to +20 HU. Because no validated quantitative threshold has been established specifically for shock-related pancreatic enhancement, the ±20 HU interval was used as a pragmatic, study-specific operational margin intended to avoid classifying small attenuation differences as biologically meaningful. It should not be interpreted as a validated diagnostic cutoff.

CT examinations were additionally reviewed for pancreatic enlargement, parenchymal edema, inflammatory peripancreatic fat stranding, acute peripancreatic fluid collections and nonenhancing pancreatic parenchyma suggestive of necrosis. Pancreatic necrosis was defined as focal or diffuse nonenhancing pancreatic tissue in the appropriate clinical and radiologic context. No included patient demonstrated pancreatic necrosis. Peripancreatic fluid alone was not considered diagnostic of acute pancreatitis.

For statistical analysis, phase-specific findings were grouped into three patient-level categories: (1) hyperenhancement/mixed hyperenhancement, defined as pancreatic hyperenhancement in at least one phase, with or without hypoenhancement in another phase; (2) hypoenhancement without hyperenhancement, defined as pancreatic hypoenhancement in at least one phase and no phase showing hyperenhancement; and (3) isoenhancement, defined as pancreatic attenuation within ±20 HU of the reference attenuation in every evaluable acquisition phase. A missing or non-evaluable delayed phase was recorded separately and was not considered evidence of isoenhancement. Patients with an unavailable delayed phase were included in the isoenhancement category only when all acquired and evaluable phases demonstrated isoenhancement.

### 2.3. Clinical Variables and Outcomes

Demographic data, main diagnosis, hospitalization duration, in-hospital outcome, time from admission to in-hospital death, and laboratory parameters were extracted from the available clinical records. Laboratory variables included erythrocyte count, leukocyte count, absolute neutrophil and lymphocyte counts, neutrophil-to-lymphocyte ratio (NLR), platelet count, glycemia, fibrinogen, C-reactive protein (CRP), procalcitonin (PCT) and albumin.

These variables were selected because they were routinely obtained during clinical care and were the laboratory parameters most consistently retrievable from the retrospective records. They were included as exploratory markers representing hematologic and coagulation abnormalities (erythrocyte count, platelet count, and fibrinogen), systemic inflammatory or infectious response (leukocyte, neutrophil and lymphocyte counts, NLR, CRP, and PCT), metabolic stress (glycemia), and general systemic illness, nutritional status, or capillary leakage (albumin). They were not intended to constitute a validated panel for grading shock severity or to substitute for established clinical severity scores. SOFA and APACHE II scores were not systematically available and could not be reliably reconstructed because several required physiological, organ-support, and time-specific variables were absent from the retrospective dataset.

Laboratory analyses were performed by complete-case analysis; zero values considered physiologically implausible for selected variables such as albumin, fibrinogen and differential percentages were treated as missing. The primary clinical outcome was all-cause in-hospital mortality during the index hospitalization. A patient was classified as deceased when death before hospital discharge was documented and as a survivor when discharge alive was documented. When neither a reliable discharge status nor an in-hospital death record could be retrieved, the outcome was classified as missing. Outcome analyses were restricted to patients with known outcomes, and no outcome values were imputed. For 14 patients, imaging and limited demographic and diagnostic information were available, but complete hospitalization records and final discharge status could not be retrieved because of unavailable discharge documentation.

### 2.4. Statistical Analysis

Continuous variables were summarized as mean ± standard deviation and/or median with interquartile range (IQR), as appropriate. Categorical variables were summarized as absolute numbers and percentages. Because of the small cohort size and the non-normal distribution of several laboratory parameters, between-group comparisons used the two-sided Mann–Whitney U test with asymptotic *p*-values and tie correction for continuous variables. Fisher’s exact test was used for 2 × 2 categorical comparisons, whereas the Fisher–Freeman–Halton exact test was used for contingency tables involving more than two categories, including the comparison of mortality across the three pancreatic enhancement groups. All exact tests were two-sided. Odds ratios (ORs) with 95% confidence intervals (CIs) were calculated for 2 × 2 outcome comparisons. A two-sided *p*-value < 0.05 was considered statistically significant. An age-adjusted analysis was evaluated for feasibility. However, only 29 patients had both known outcome and available age, comprising 19 deaths and 10 survivors, and the resulting two-predictor logistic model produced a highly unstable estimate consistent with sparse-data bias. Albumin was available for only 22 patients with known outcomes, and the individual shock subgroups contained insufficient numbers for reliable additional adjustment. Furthermore, SOFA and APACHE II severity scores, vasopressor dose, lactate level, mechanical ventilation, fluid-resuscitation volume, cardiac output, and the timing of hemodynamic deterioration relative to CT were not systematically available. Consequently, no adjusted effect estimate was used for inference, and all reported mortality associations were considered exploratory, unadjusted complete-case analyses.

All revised statistical analyses were performed using Python version 3.12.13 (Python Software Foundation, Wilmington, DE, USA), with NumPy version 2.3.5 and SciPy version 1.17.0. Fisher–Freeman–Halton’s exact *p*-values for 2 × 3 contingency tables were obtained by exhaustive enumeration of all possible tables with fixed marginal totals.

To assess potential selection bias related to missing outcome information, patients with and without available outcomes were compared with respect to age, sex, recorded shock category and pancreatic enhancement pattern. The Mann–Whitney U test was used for age, Fisher’s exact test for binary variables and an exact test for the overall three-category enhancement distribution. These comparisons were exploratory, and no adjustment for multiple testing was performed.

## 3. Results

### 3.1. Study Population and Outcomes

During the study period, 266 ICU patients with a clinically established diagnosis of shock were identified using the predefined ICD-10 codes. Of these, 94 underwent abdominal CT and were assessed for imaging eligibility. Fifty did not fulfill the predefined imaging criteria for the CT hypoperfusion complex. The final analytical cohort therefore consisted of 44 unique patients, each represented by one eligible CT examination (Figure 1). Age was available for 43 patients; mean age was 59.5 ± 21.2 years, median age was 66 years (IQR 49–76; range 15–86 years). The cohort included 28 women (63.6%) and 16 men (36.4%). Clinical contexts were heterogeneous and included traumatic/hemorrhagic-hypovolemic shock patterns (17/44, 38.6%), septic or infectious conditions (8/44, 18.2%), cardiogenic shock (7/44, 15.9%), abdominal surgical/ischemic conditions (5/44, 11.4%) and other or unspecified shock contexts (12/44, 27.3%). These diagnostic categories were not mutually exclusive.

Outcome data were available for 30 patients (68.2%). Among these patients, 20 died, corresponding to an observed mortality of 66.7% (95% CI 48.8–80.8%). Median hospitalization duration in the outcome-available group was 6.0 days (IQR 3.0–18.0; range 2 h–68 days). Among patients who died, median time from admission to death was 4.5 days (IQR 2.0–10.5; range 2 h–42 days); eight of the 20 deaths (40.0%) occurred within 72 h. See Table 1.

No patient included in the final cohort had a confirmed clinical or radiologic diagnosis of acute pancreatitis, and no pancreatic necrosis was identified.

In-hospital outcome information was available for 30/44 patients (68.2%) and was missing for 14/44 patients (31.8%). Among patients with known outcomes, 20/30 died during the index hospitalization (66.7%), and 10/30 were discharged alive (33.3%). This proportion represents mortality only among patients with documented outcomes and should not be interpreted as the mortality rate of the entire cohort.

Patients with and without available outcomes did not differ significantly in age or sex. However, the other/unspecified shock designation was more frequent among patients with missing outcomes (8/14, 57.1%) than among those with known outcomes (4/30, 13.3%; Fisher’s exact *p* = 0.004). The pancreatic enhancement distribution also differed between the groups: hyperenhancement/mixed enhancement, hypoenhancement without hyperenhancement, and isoenhancement were present in 10/30, 8/30, and 12/30 patients with known outcomes, respectively, compared with 10/14, 3/14 and 1/14 patients with missing outcomes (exact *p* = 0.033). Any abnormal pancreatic enhancement was present in 18/30 patients (60.0%) with known outcomes and 13/14 patients (92.9%) with missing outcomes (Fisher’s exact *p* = 0.035). These differences indicate that outcome missingness may not have been random.

A detailed comparison of patients with available and missing outcome data is provided in Table 2.

The overall comparison of mortality across the three mutually exclusive pancreatic enhancement categories was performed using the two-sided Fisher–Freeman–Halton exact test (*p* = 0.064). Mortality analyses included only the 30 patients with known in-hospital outcomes.

### 3.2. Pancreatic Enhancement Patterns

Abnormal pancreatic enhancement was documented in 31/44 patients (70.5%; 95% CI 55.8–81.8%). Hyperenhancement or mixed hyperenhancing patterns were present in 20/44 patients (45.5%), hypoenhancement without any hyperenhancing phase was present in 11/44 patients (25.0%), and 13/44 patients (29.5%) demonstrated isoenhancement in all evaluable phases. Peripancreatic fluid was recorded in two cases; both were classified in the hypoenhancement-without-hyperenhancement group.

All 13 patients assigned to the isoenhancement category had an explicit phase-by-phase pancreatic assessment. No patient was classified as isoenhancing solely because hyperenhancement or hypoenhancement was not documented. Missing delayed-phase acquisitions were analyzed separately as not acquired/not evaluable.

Phase-specific analysis showed that the arterial phase most frequently demonstrated isoenhancement (22/44, 50.0%), followed by hyperenhancement (15/44, 34.1%) and hypoenhancement (7/44, 15.9%). In the portal venous phase, isoenhancement was seen in 20/44 cases (45.5%), hypoenhancement in 14/44 (31.8%) and hyperenhancement in 10/44 (22.7%). In the delayed phase, isoenhancement was most frequent (28/44, 63.6%); hyperenhancement and hypoenhancement were each identified in six cases (13.6%), while the delayed phase was not evaluable or not acquired in four cases (9.1%). See the Table 3 and Table 4.

### 3.3. Enhancement Pattern and Mortality

Among the 30 patients with available outcome data, death occurred in 9/10 patients (90.0%) with hyperenhancement/mixed hyperenhancement, 6/8 patients (75.0%) with hypoenhancement without hyperenhancement, and 5/12 patients (41.7%) with isoenhancement. The overall difference in mortality across the three pancreatic enhancement categories did not reach statistical significance using the two-sided Fisher–Freeman–Halton exact test (*p* = 0.064). Therefore, the group-specific mortality proportions should be interpreted descriptively, and the limited subgroup sizes and non-significant global comparison preclude a definitive conclusion that the hyperenhancement/mixed phenotype indicates the worst prognosis.

In an exploratory, unadjusted binary complete-case analysis, death before hospital discharge occurred in 15/18 patients (83.3%) with abnormal pancreatic enhancement and in 5/12 patients (41.7%) with isoenhancement (Fisher’s exact *p* = 0.045; OR 7.0, 95% CI 1.29–37.91). In a separate exploratory comparison, death before hospital discharge occurred in 9/10 patients (90.0%) with pancreatic hyperenhancement in at least one phase and in 11/20 patients (55.0%) without hyperenhancement (Fisher’s exact *p* = 0.101; OR 7.36, 95% CI 0.78–69.58). Both analyses were restricted to patients with known in-hospital outcomes and were not adjusted for age, shock subtype, disease severity, or other potential confounders. Laboratory data completeness varied by parameter. Among the 30 patients with known outcomes, erythrocyte, leukocyte, neutrophil, lymphocyte, NLR, platelet, and glycemia values were available for all patients. After physiologically implausible zero values were treated as missing, evaluable data were available for 27 patients for fibrinogen, 29 for CRP, 20 for PCT, and 22 for albumin. All laboratory comparisons were complete-case analyses of exploratory nature.

Non-survivors were older than survivors (median age 66.0 vs. 45.5 years; *p* = 0.023) and had lower albumin values in the complete-case laboratory analysis (median 2.43 vs. 3.05; *p* = 0.017). Leukocyte count, neutrophil count, NLR, platelet count, glycemia, CRP and PCT did not show statistically significant differences between survivors and non-survivors in this cohort.

Age was available for 29 of the 30 patients with known outcomes, whereas albumin was available for 22 patients. Because of the limited number of complete observations and the sparse outcome distribution, a reliable adjusted effect estimate could not be obtained. Accordingly, the reported associations between pancreatic enhancement and mortality are unadjusted and exploratory.

### 3.4. Representative Imaging Findings:

**Figure 2:**
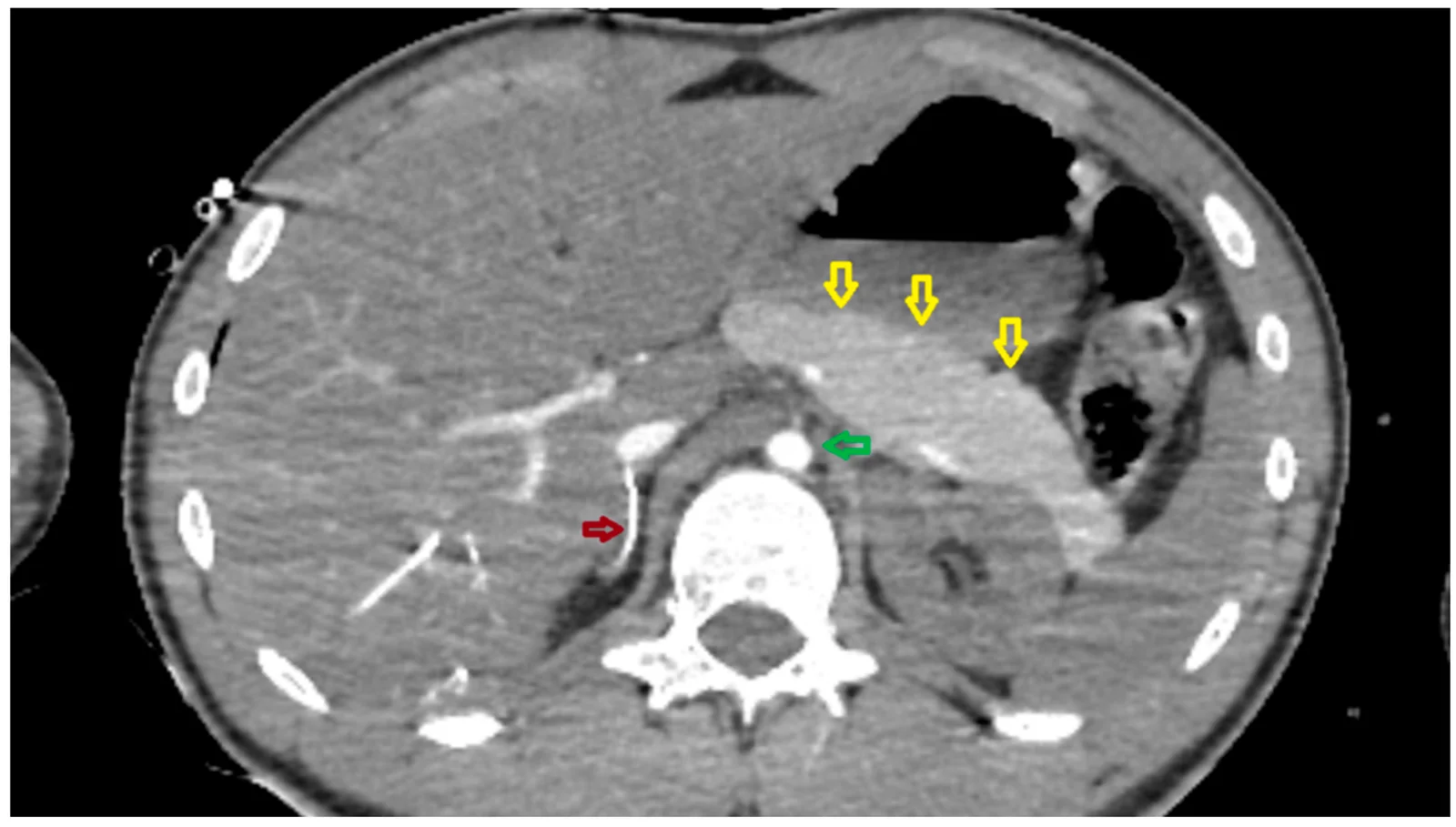
Hyperenhancing pancreatic parenchyma (yellow arrows); diminished abdominal aortic caliber (green arrow); and hyperenhancing adrenal gland (red arrow).

**Figure 3:**
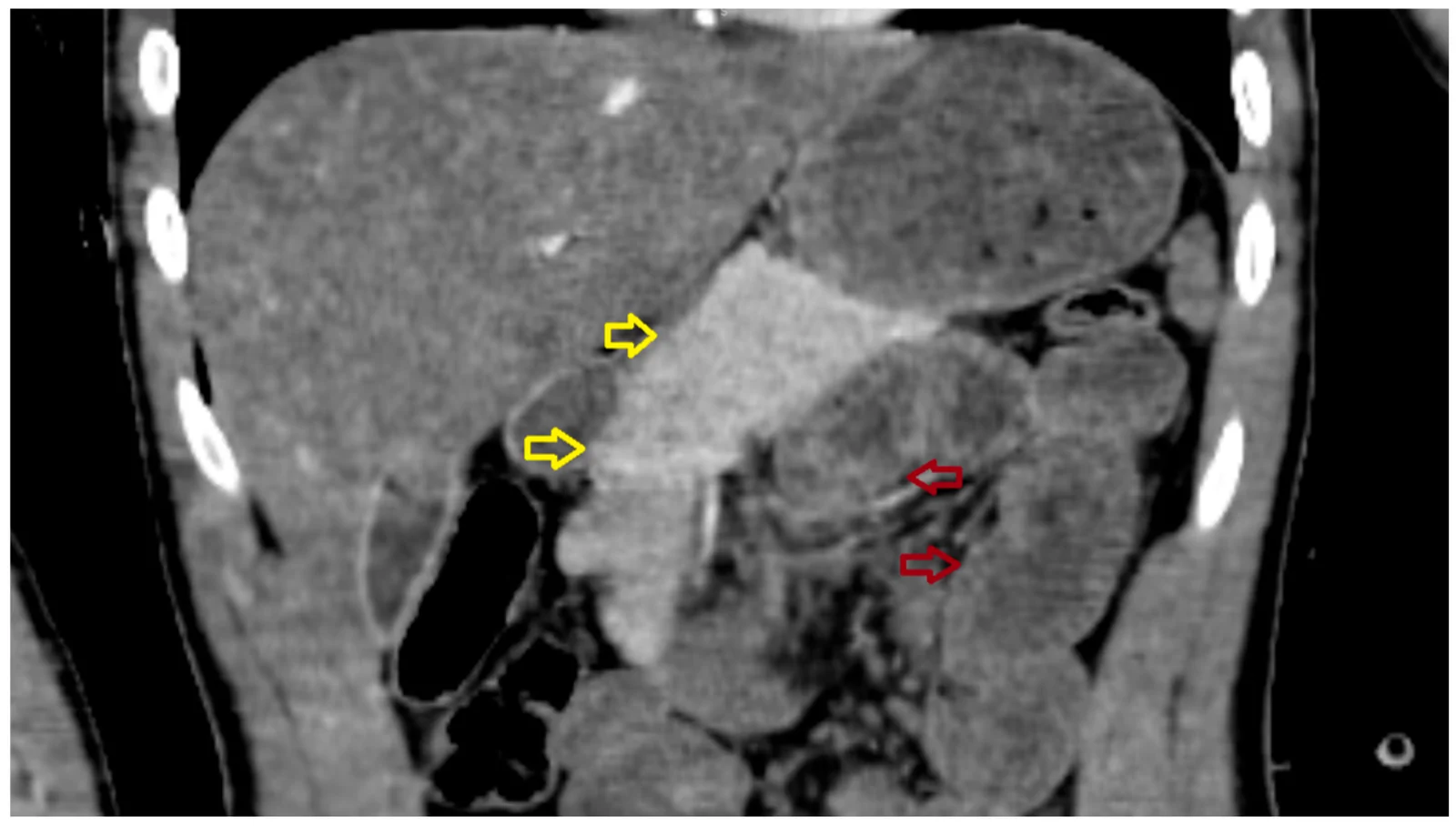
Hyperenhancing pancreatic parenchyma (yellow arrows) associated with thickened small-bowel wall and hyperenhancing mucosa caused by submucosal edema (red arrows).

**Figure 4:**
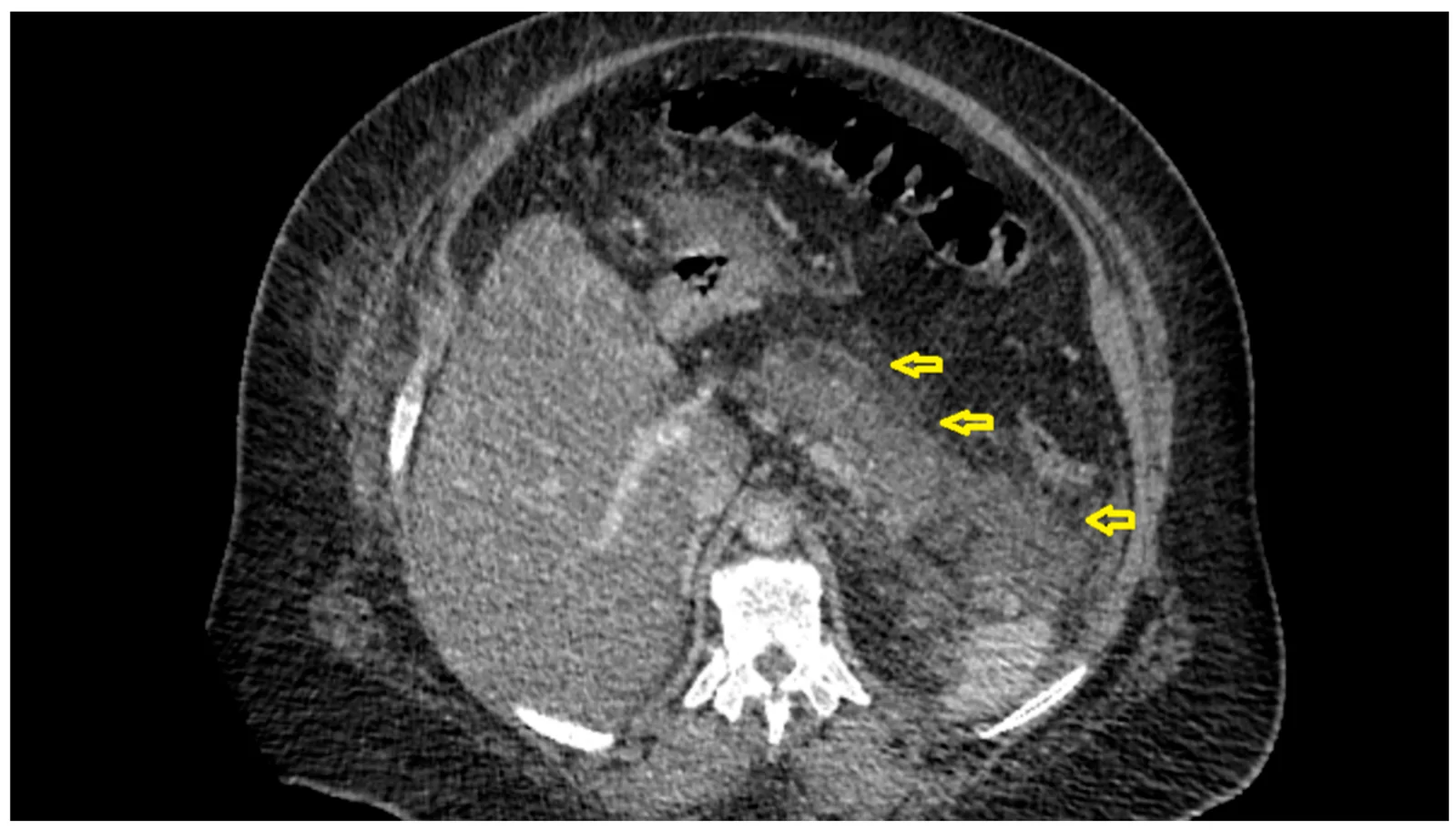
Hypoenhancing pancreatic parenchyma with peripancreatic fluid (yellow arrows).

**Figure 5:**
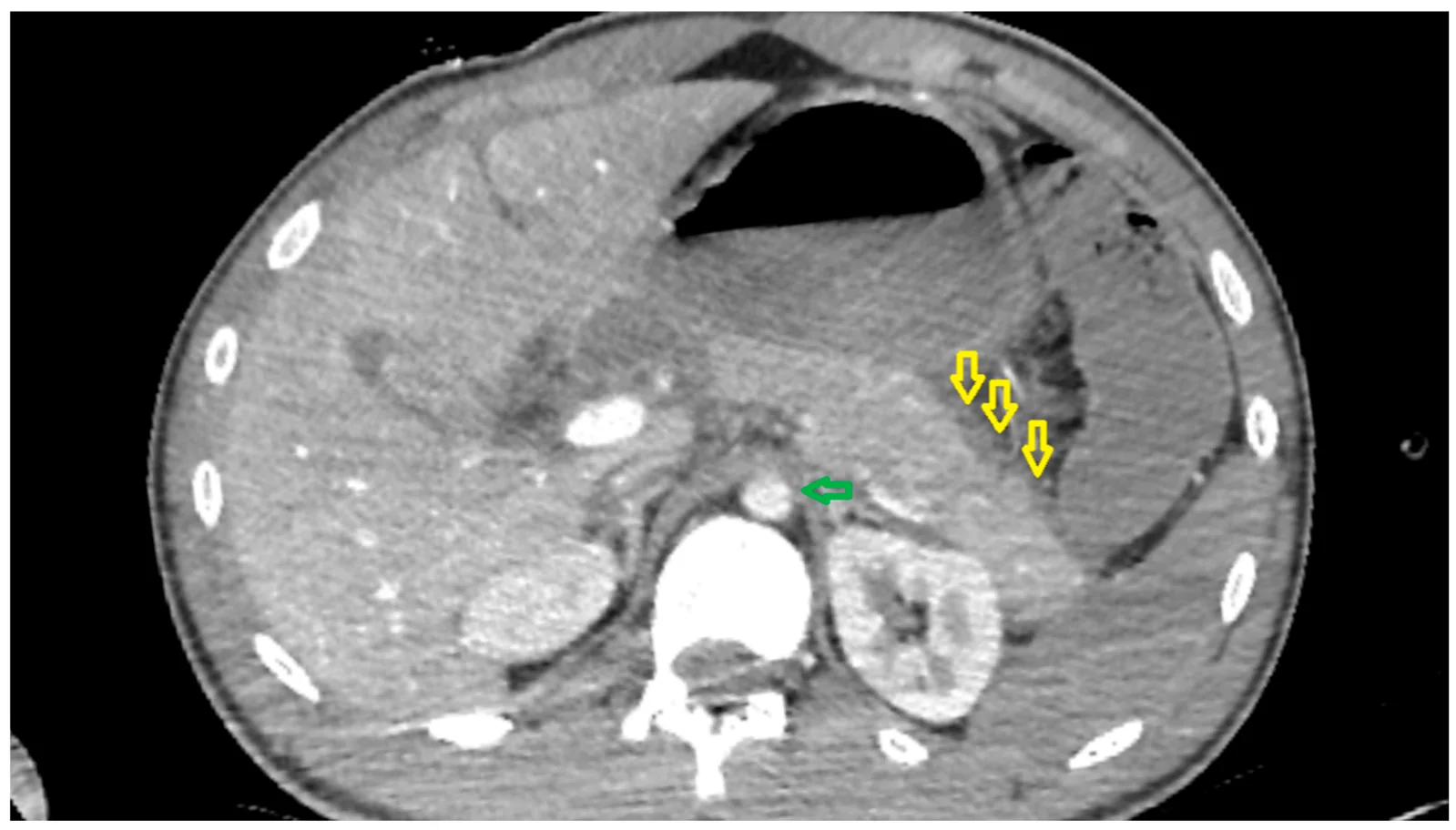
Peripancreatic fluid (yellow arrows) with isoenhancing pancreatic parenchyma and small-caliber abdominal aorta (green arrows).

Representative imaging findings suggestive of pancreatic changes in the setting of shock are presented in Figure 2, Figure 3, Figure 4 and Figure 5.

## 4. Discussion

This retrospective cohort showed that pancreatic enhancement variability was frequent among ICU patients with CT hypoperfusion complex. Abnormal pancreatic enhancement was identified in 31 of the 44 patients (70.5%). Hyperenhancement or mixed hyperenhancement was the most common abnormal patient-level pattern, while a substantial proportion of patients showed hypoenhancement without a hyperenhancing phase.

The divergent pancreatic appearances observed in this study are consistent with the conflicting literature. Taylor et al. and Sivit et al. described intense pancreatic enhancement as part of the classic pediatric post-traumatic hypoperfusion complex [1,7]. Adult reports later emphasized peripancreatic fluid and variable pancreatic enhancement, and Ryan et al. specifically described peripancreatic fluid as a useful sign of the hypovolemic shock complex in adults [8]. Higashi et al. added an important dynamic perspective by suggesting that pancreatic perfusion may decrease in the early phase while delayed or prolonged pancreatic enhancement may be seen later [9]. Our findings support this heterogeneity: arterial hyperenhancement, portal venous hypoenhancement, and delayed hyper- or hypoenhancement were all present across the cohort.

The pathophysiology of shock pancreas remains incompletely clarified. In systemic hypotension, sympathetic activation and centralization of blood flow are expected to cause splanchnic vasoconstriction. This mechanism explains shock bowel and visceral hypoenhancement but does not fully explain intense pancreatic hyperenhancement. Proposed explanations include transient redistribution of contrast, altered microvascular permeability, changes in interstitial pressure, delayed washout and timing-related effects of multiphasic CT in patients with profoundly abnormal circulation [5,9,15]. The mixed patterns observed in several patients in the present cohort support the idea that pancreatic enhancement is not a binary sign but a dynamic phenomenon influenced by phase timing, organ perfusion and capillary leak.

An exploratory unadjusted association was observed between abnormal pancreatic enhancement and all-cause in-hospital mortality among patients with known outcomes. The wide confidence interval around the exploratory binary odds ratio (95% CI 1.29–37.91) further indicates substantial statistical imprecision. However, the global exact comparison across the three pancreatic enhancement categories did not reach statistical significance (Fisher–Freeman–Halton exact *p* = 0.064), and the category-specific mortality proportions should therefore be interpreted descriptively. The binary complete-case association between abnormal enhancement and mortality does not establish causality or independent prognostic value. Because clinical and biological evidence of shock preceded CT examination, abnormal pancreatic perfusion is more plausibly a consequence of severe systemic hemodynamic compromise than a direct contributor to death. It may therefore represent an imaging correlate of the severity of the broader CT hypoperfusion complex. In addition, non-survivors were older and had lower albumin levels; shock etiologies and underlying diseases were heterogeneous, and several major indicators of disease severity were unavailable. Residual confounding and the potentially non-random absence of outcome information in 14 patients further limit interpretation of this association.

Our mortality rate among patients with available outcome data was high, which is compatible with the known severity of CT shock patterns. Contemporary reviews emphasize that CT signs of shock and imminent circulatory collapse are prognostically important imaging biomarkers and should prompt urgent communication with the treating team [15,16]. In the trauma-focused literature, recognition of the hypovolemic shock complex is considered clinically useful because secondary CT signs may reveal the severity of hemodynamic compromise even when CT is primarily requested to identify the source of hemorrhage [17]. The present ICU cohort extends this principle to mixed traumatic and non-traumatic shock contexts.

Recent studies in non-traumatic undifferentiated shock have also shown that contrast-enhanced thoraco-abdominal CT can support etiologic classification and clinical management, while a greater burden of CT hypoperfusion-complex signs was associated with higher observed mortality [18,19].

The global exact comparison of mortality across the three pancreatic enhancement phenotypes did not reach statistical significance (Fisher–Freeman–Halton exact *p* = 0.064), and the mortality proportions of the individual categories should therefore be interpreted descriptively. In the exploratory binary complete-case analysis, abnormal pancreatic enhancement was associated with mortality. However, non-survivors were significantly older and had lower serum albumin levels, while shock etiologies and underlying diseases were highly heterogeneous. Moreover, several important indicators of disease severity and resuscitation, including vasopressor dose, lactate level, mechanical ventilation, fluid-resuscitation volume, cardiac output, and validated shock-severity scores, were unavailable or incomplete. Consequently, residual confounding is highly probable, and the observed association should not be interpreted as evidence that pancreatic enhancement independently predicts mortality. Pancreatic enhancement may instead represent one component of the broader imaging phenotype of severe systemic hemodynamic compromise.

Several limitations should be acknowledged. First, this was a retrospective single-center study with a limited sample size. Second, outcome and laboratory data were not available for all 44 patients; mortality analyses were restricted to 30 patients. Third, ROI-based pancreatic measurements were performed relative to liver and spleen, organs that may themselves show abnormal perfusion in shock. Fourth, CT acquisition timing and delayed-phase availability varied according to clinical feasibility. Fifth, SOFA and APACHE II scores and other important indicators of shock severity, including vasopressor dose, lactate level, mechanical ventilation, fluid-resuscitation volume, cardiac output, renal function, and the interval between hemodynamic deterioration and CT, were not systematically available. Consequently, a reliable adjusted analysis was not feasible, and residual confounding may account for part of the observed association between pancreatic enhancement and mortality. The retrospective design also precludes determination of causal direction. Because shock was clinically and biologically established before CT examination, abnormal pancreatic perfusion may primarily represent a consequence of severe systemic hemodynamic compromise rather than an independent determinant of mortality. Therefore, the observed association cannot be interpreted as evidence that abnormal pancreatic enhancement causes death or independently predicts outcome. Finally, zero values in some laboratory fields appeared to represent non-measured or physiologically implausible entries and were handled as missing for selected comparisons. Additionally, clinical shock identification relied on the diagnosis established by the treating team and the corresponding ICD-10 coding, supported by the routinely available clinical, laboratory, and hemodynamic information, rather than on a single uniform study-specific numerical threshold. Consequently, some degree of retrospective clinical classification bias cannot be excluded.

An important additional limitation is that pancreatic enhancement was defined relative to hepatic and splenic attenuation rather than by an absolute or perfusion-based measurement. Both reference organs may themselves demonstrate altered enhancement during systemic hypoperfusion. Consequently, hepatic or splenic hypoenhancement may exaggerate the apparent relative hyperenhancement of the pancreas, whereas abnormal enhancement of the reference organs may mask pancreatic hypoenhancement. The resulting categories should therefore be interpreted as relative organ-to-organ enhancement phenotypes and not as direct quantitative measurements of pancreatic perfusion. Because the degree of reference-organ hypoperfusion may also reflect shock severity, this measurement bias could influence the observed association between pancreatic enhancement category and mortality. Future prospective studies should use standardized phase-specific absolute pancreatic attenuation measurements and, where feasible, CT perfusion parameters, rather than relying exclusively on relative pancreas-to-liver or pancreas-to-spleen attenuation differences.

Recent multiphase dual-energy CT research has demonstrated the feasibility of quantitative pancreatic iodine measurements and has identified patient-related factors that influence pancreatic iodine uptake, further supporting the need for standardized quantitative protocols [20].

Although patients with confirmed acute pancreatitis or pancreatic necrosis were excluded, pancreatic enzyme measurements were not systematically available for every patient because of the retrospective design. Therefore, clinically occult or incompletely documented pancreatic inflammation cannot be excluded with absolute certainty. Nevertheless, none of the included patients had a documented clinical diagnosis or characteristic CT findings of acute pancreatitis, and peripancreatic fluid was interpreted as part of the hypoperfusion complex only in the absence of additional evidence of pancreatic inflammation.

A major limitation is that in-hospital outcome information was unavailable for 14/44 patients (31.8%). Patients with missing outcomes differed from those with known outcomes in both recorded shock category and pancreatic enhancement distribution, indicating that the missingness may not have been random. In particular, abnormal pancreatic enhancement was more frequent among patients with missing outcomes. Therefore, the direction and magnitude of the potential selection bias cannot be reliably determined, and the observed association between pancreatic enhancement and mortality applies only to the complete-case subset. No mortality rate was calculated for the entire cohort, and no missing outcomes were imputed.

The small number of patients with known outcomes and the sparse distribution of survivors across enhancement categories precluded a reliable adjusted analysis. Although age adjustment was assessed, the resulting estimate was unstable and was not considered suitable for inference. Additional adjustment for albumin, shock subtype, baseline disease severity, vasopressor use, lactate level, mechanical ventilation, and fluid resuscitation was not feasible because these variables were either incomplete, unavailable, or represented by very small subgroups. Residual confounding therefore remains a major limitation, and no independent or causal prognostic role can be attributed to pancreatic enhancement. This limitation is compounded by the potentially non-random absence of outcome information for 14 patients.

Despite these limitations, the findings support structured assessment of the pancreas when CT hypoperfusion complex is suspected. Radiology reports should describe pancreatic enhancement phase by phase and should avoid assuming that shock pancreas always appears as hyperenhancement. A practical reporting approach is to state whether pancreatic enhancement is hyperenhancing, hypoenhancing, mixed or isoenhancing relative to the liver/spleen and to mention associated peripancreatic fluid or other hypoperfusion signs. Future prospective studies should incorporate standardized contrast timing, quantitative perfusion measures, serial CT or clinical follow-up, vasopressor and lactate data, and multivariable mortality models.

Future prospective studies with larger cohorts and standardized recording of the interval between CT examination and death should separately evaluate early mortality, defined as death within 72 h after CT, and later in-hospital mortality across pancreatic enhancement phenotypes.

## 5. Conclusions

Pancreatic enhancement variability is a common finding in ICU patients with CT hypoperfusion complex. In this 44-case retrospective cohort, abnormal pancreatic enhancement was present in 70.5% of examinations and included hyperenhancing, hypoenhancing and mixed phase-specific patterns.

Among patients with known outcomes, abnormal pancreatic enhancement was associated with all-cause in-hospital mortality in an exploratory, unadjusted complete-case analysis. However, because of missing outcome data, limited sample size, heterogeneous shock etiologies, and the inability to adjust reliably for age and other indicators of disease severity, no causal or independent prognostic role can be inferred. Because shock was clinically and biologically established before CT examination, pancreatic enhancement abnormalities are more plausibly interpreted as imaging correlates of severe systemic hemodynamic compromise than as determinants of mortality. They should therefore be interpreted as part of the broader CT hypoperfusion complex rather than as independent predictors of outcome.

## Figures and Tables

**Table 1:** Baseline clinical and laboratory variables in the outcome-available cohort.

Variable	Overall	Died	Survived	*p*-Value
Age, years	64.0 (44.0–72.0); *n* = 29	66.0 (58.0–76.0); *n* = 19	45.5 (27.0–61.8); *n* = 10	0.023
Female sex	18/30 (60.0%)	12/20 (60.0%)	6/10 (60.0%)	1.000
Hospitalization, days	6.0 (3.0–18.0); *n* = 30	4.5 (2.0–10.5); *n* = 20	12.5 (6.0–23.3); *n* = 10	0.058
Erythrocytes, ×10^12^/L	3.95 (3.45–4.43); *n* = 30	4.10 (3.70–4.59); *n* = 20	3.48 (3.09–3.95); *n* = 10	0.061
Leukocytes, ×10^9^/L	14.20 (9.55–20.58); *n* = 30	15.26 (8.63–19.71); *n* = 20	13.73 (10.45–20.46); *n* = 10	0.912
Neutrophils, ×10^9^/L	12.09 (8.00–16.99); *n* = 30	11.20 (7.17–16.80); *n* = 20	12.09 (9.07–16.30); *n* = 10	0.912
Lymphocytes, ×10^9^/L	1.18 (0.69–1.83); *n* = 30	1.18 (0.81–1.56); *n* = 20	1.17 (0.66–2.01); *n* = 10	0.982
NLR, dimensionless ratio	9.22 (6.54–13.32); *n* = 30	9.88 (7.49–12.94); *n* = 20	8.68 (6.12–13.78); *n* = 10	0.912
Platelets, ×10^9^/L	251.2 (186.1–345.5); *n* = 30	251.2 (187.8–374.8); *n* = 20	256.4 (184.0–294.3); *n* = 10	0.741
Blood glucose, mg/dL	164.5 (98.0–217.8); *n* = 30	171.0 (113.8–245.0); *n* = 20	158.0 (85.3–206.0); *n* = 10	0.391
Fibrinogen, mg/dL	401.0 (184.0–606.5); *n* = 27	531.0 (320.0–660.0); *n* = 17	243.5 (162.0–397.8); *n* = 10	0.093
CRP, mg/L	71.8 (22.8–358.8); *n* = 29	75.1 (34.0–355.0); *n* = 19	51.5 (25.1–309.8); *n* = 10	0.477
PCT, ng/mL	2.04 (0.90–5.26); *n* = 19	1.98 (0.64–2.06); *n* = 12	5.25 (2.87–5.28); *n* = 7	0.056
Albumin, g/dL	2.49 (2.30–3.25); *n* = 22	2.43 (2.23–2.52); *n* = 14	3.05 (2.65–3.63); *n* = 8	0.017

Values are median (IQR) unless otherwise specified. Outcome data were available for 30 patients. Laboratory analyses used complete cases; zero values judged physiologically implausible for selected laboratory fields were treated as missing. NLR: neutrophil-to-lymphocyte ratio; CRP: C-reactive protein; PCT: procalcitonin. For continuous-variable comparisons, *p*-values were calculated using the two-sided asymptotic Mann–Whitney U test with tie correction and were rounded to three decimal places.

**Table 2:** Patients with available and missing outcome data.

Characteristic	Outcome Available, *n* = 30	Outcome Missing, *n* = 14	*p*-Value
Age, years, median (IQR)	64.0 (44.0–72.0), *n* = 29	69.5 (59.3–76.0), *n* = 14	0.204
Female sex	18/30 (60.0%)	10/14 (71.4%)	0.521
Cardiogenic shock code	5/30 (16.7%)	2/14 (14.3%)	1.000
Hypovolemic shock code	5/30 (16.7%)	5/14 (35.7%)	0.247
Septic shock code	4/30 (13.3%)	0/14 (0.0%)	0.290
Other/unspecified shock code	4/30 (13.3%)	8/14 (57.1%)	0.004
Hyperenhancement/mixed pattern	10/30 (33.3%)	10/14 (71.4%)	0.033 *
Hypoenhancement without hyperenhancement	8/30 (26.7%)	3/14 (21.4%)	—
Isoenhancement in all evaluable phases	12/30 (40.0%)	1/14 (7.1%)	—
Any abnormal pancreatic enhancement	18/30 (60.0%)	13/14 (92.9%)	0.035

* *p*-value for the overall three-category enhancement distribution. Shock-code categories were not mutually exclusive. Age was compared using the Mann–Whitney U test; binary variables were assessed using Fisher’s exact test.

**Table 3:** Patient-level pancreatic enhancement patterns and mortality.

Enhancement Category	All Patients	Outcome Available	Deaths in Category	Exploratory Association with Mortality
Hyperenhancement/mixed hyperenhancement	20/44 (45.5%)	10/30 (33.3%)	9/10 (90.0%)	Reference subgroup for descriptive comparison
Hypoenhancement without hyperenhancement	11/44 (25.0%)	8/30 (26.7%)	6/8 (75.0%)	Fisher *p* = 0.682 vs. all others
Iso/no abnormal enhancement documented	13/44 (29.5%)	12/30 (40.0%)	5/12 (41.7%)	Reference for abnormal vs. no abnormal
Any abnormal pancreatic enhancement	31/44 (70.5%)	18/30 (60.0%)	15/18 (83.3%)	OR 7.0; 95% CI 1.29–37.91; Fisher *p* = 0.045
Any pancreatic hyperenhancement	20/44 (45.5%)	10/30 (33.3%)	9/10 (90.0%)	OR 7.36; 95% CI 0.78–69.58; Fisher *p* = 0.101
Any pancreatic hypoenhancement	15/44 (34.1%)	11/30 (36.7%)	10/11 (90.9%)	Non-exclusive; includes mixed cases

Hyperenhancement/mixed hyperenhancement: at least one hyperenhancing phase. Hypoenhancement without hyperenhancement: at least one hypoenhancing phase and no hyperenhancing phase. The last row is non-exclusive because some mixed cases had both hyperenhancing and hypoenhancing phases.

**Table 4:** Phase-specific pancreatic enhancement distribution.

CT Phase	Hyperenhancement	Isoenhancement	Hypoenhancement	Not Evaluable/Not Acquired
Arterial phase	15/44 (34.1%)	22/44 (50.0%)	7/44 (15.9%)	0/44 (0.0%)
Portal venous phase	10/44 (22.7%)	20/44 (45.5%)	14/44 (31.8%)	0/44 (0.0%)
Delayed phase	6/44 (13.6%)	28/44 (63.6%)	6/44 (13.6%)	4/44 (9.1%)

Enhancement categories were defined relative to liver and/or spleen attenuation in the same acquisition phase using a ±20 HU threshold.

## Data Availability

The original contributions presented in this study are included in the article. Further inquiries can be directed to the corresponding author.
